# First‐trimester preexisting diabetes screening in Medicaid beneficiaries

**DOI:** 10.1002/pmf2.70290

**Published:** 2026-04-09

**Authors:** Zeinab Kassem, Sebastian Z. Ramos, Benjamin Koethe, Mohak Mhatre

**Affiliations:** ^1^ Division of Maternal Fetal Medicine Department of Obstetrics and Gynecology Tufts University School of Medicine Boston Massachusetts USA; ^2^ BERD Center Clinical and Translational Science Institute Tufts University School of Medicine Boston Massachusetts USA

**Keywords:** diabetes mellitus, health disparities, high‐risk pregnancy, prenatal care, preventative care, public health insurance, screening

## Abstract

**Introduction:**

Patients with public insurance have higher rates of pregestational diabetes mellitus (DM) and are less likely to receive preconception care than those with private insurance. First trimester DM screening rates among high‐risk publicly insured pregnant individuals in the United States are unknown. This study assessed first‐trimester DM screening rates in this population. We hypothesized that less than half of publicly insured pregnant patients with at least one DM risk factor were screened in the first trimester. We also examined the utilization of different DM screening tests and pregnancy outcomes between those who did and did not receive early screening.

**Methods:**

This retrospective cohort study used US claims‐based data from January 2020 to December 2022. Publicly insured individuals with a viable intrauterine pregnancy, who presented for care before 14 weeks, and had no history of DM were included. International classification of diseases 10th revision codes identified patients who had at least one risk factor for DM. Current procedural terminology codes identified DM screening tests. Chi‐square test compared categorical variables, and two‐sample *t*‐test compared continuous variables. Logistic regression assessed risk factors associated with early DM screening.

**Results:**

A total of 240,109 individuals met inclusion criteria. Among them, 64,230 patients had at least one DM risk factor of which 27,059 (42%) underwent early screening and 37,171 (58%) did not. Insulin resistance (adjusted odds ratio [aOR] 9.47, 95% confidence interval [CI] 8.58–10.47), history of gestational diabetes (aOR 5.89, 95% CI 5.4–6.43), family history of DM (aOR 3.34, 95% CI 3–3.71), prepregnancy obesity (aOR 3.17, 95% CI 3.04–3.31), and hyperlipidemia (aOR 1.59, 95% CI 1.41–1.8) were most strongly associated with early DM screening. Compared to high‐risk patients who were not screened, those who were screened had higher rates of gestational hypertension (21.2% vs. 19.7%, *p* < 0.001), preeclampsia (17.4% vs. 15.6%, *p* < 0.001), and gestational diabetes (22.8% vs. 12.9%, *p* < 0.001). Hemoglobin A1c and 1‐h oral glucose tolerance test were the most used screening tests (50.0% and 29.4%, respectively).

**Conclusions:**

More than 50% of publicly insured individuals with at least one DM risk factor did not undergo first trimester DM screening, highlighting a potential missed opportunity for screening a high risk population.

## 1 INTRODUCTION

Diabetes mellitus (DM) imposes a burden on pregnant individuals and is associated with health outcomes affecting the birthing parent/fetus dyad, including cardiovascular disease (CVD), hypertension, venous thromboembolism, and neuropathy as well as increased risk of obesity and impaired glucose metabolism in the offspring [1, 2, 3]. In 2019, the prevalence of type 2 diabetes among reproductive‐aged women in the United States was 4.5%, with 30% unaware of their diagnosis and half of those diagnosed with having uncontrolled disease [4]. Moreover, global data showed a sustained increase in DM among this population, with incidence and disease burden doubling since 1990, and a projected threefold rise by 2045 [5, 6]. Because uncontrolled diabetes in early pregnancy increases the risk of severe congenital anomalies, preconception diagnosis and management are essential [7]. Preconception care for patients with known pregestational DM could save up to $4.3 billion in neonatal health‐care costs, with another $1.2 billion averted among patients with undiagnosed DM [8].

Screening for diabetes in the first trimester of pregnancy is recommended in individuals at risk for developing DM who have obesity plus another high risk factor [9]. However, this risk‐based approach assumes accurate identification of multiple risk factors prior to or early in pregnancy, which may not occur in populations with limited access to preconception care. Abnormal glucose metabolism and undiagnosed DM are common in reproductive‐aged women and clinically significant hyperglycemia may be present even in individuals who do not meet strict screening criteria [4, 10]. Reliance on more restrictive screening thresholds may therefore miss a substantial proportion of patients with DM [11].

In the United States, 40% of pregnancies are covered by public insurance as compared to 55%, which are covered by private insurance [12]. These coverage patterns underscore the importance of evaluating screening practices within publicly insured populations. In addition, publicly insured patients have higher DM rates and are less likely to receive preconception care than those with private insurance [13, 14, 15]. Early detection of pregestational DM is therefore critical to improving outcomes and reducing costs. Among nonpregnant individuals eligible for screening based on American Diabetes Association or United States Preventive Services Task Force recommendations, approximately 50% undergo diabetes screening [16, 17]. A recent study of privately insured pregnant individuals in the United States found first‐trimester DM screening rates of approximately 20% [18]. The rate for publicly insured patients with at least one diabetes risk factor is currently unknown. Given the well‐documented disparities in preventive care access among publicly insured cohorts, the primary objective of this study was to evaluate the early screening rate, with the hypothesis that fewer than half of these patients undergo early screening.

## 2 MATERIALS AND METHODS

This was a retrospective cohort study of pregnant individuals with public insurance in the United States from January 2020 to December 2022 using the Merative MarketScan database. This is a large, nationwide, de‐identified, health‐care claims database with patient‐level data on health‐care utilization, costs, and outcomes, including inpatient, outpatient, and pharmacy claims. Although the database includes multiple insurance types, our analysis focused on Medicaid claims only. As the dataset was de‐identified, this study was exempt by our institutional review board.

The primary outcome was the rate of first‐trimester diabetes screening among publicly insured pregnant patients who had at least one DM risk factor. Secondary outcomes included screening rates by race, predictors of early screening, pregnancy outcomes between screened and unscreened high‐risk individuals, and frequency of diagnostic test use. International classification of diseases 10th edition (ICD‐10) codes were used to identify patients aged 15–44 years with a live intrauterine pregnancy <14 weeks gestational age (see Supporting Information Appendix A). Patients were excluded if they had nonviable pregnancies (missed, incomplete, or spontaneous abortion, and molar or ectopic pregnancy), pregestational diabetes (type 1 DM and type 2 DM), or pregnancies ≥ 14 weeks gestation. The algorithm developed by Ailes et al. for MarketScan was used to identify the cohort [19]. Each pregnancy was treated as an independent analytic event. Although a small proportion of individuals contributed more than one pregnancy during the study period, more than 90% of pregnancies represented the first observable pregnancy in the dataset. Given this small proportion and dataset constraints, clustering by individual was not performed.

ICD‐10 codes were also used to identify risk factors for diabetes, including prepregnancy body mass index ≥ 30 kg/m^2^, history of gestational diabetes mellitus (GDM), hypertension, family history of DM, polycystic ovary syndrome (PCOS), hyperlipidemia, history of macrosomia or large for gestational age (LGA) infant, physical inactivity, insulin resistance, CVD, and human immunodeficiency virus (HIV) (Supporting Information Appendix B). Additionally, we included social determinants of health (SDOH) (Supporting Information Appendix C). A patient was defined as “high risk” if they had at least one of these risk factors. Demographics included age and race/ethnicity as classified in the dataset (White, Black, Hispanic, other, unknown). Geographic information was unavailable. Screening tests were identified using current procedural terminology (CPT) codes for hemoglobin A1C (HgbA1c), oral glucose tolerance tests (GTTs) (50, 75, and 100 g; 1, 2, and 3 h), fasting plasma glucose, and random fingerstick glucose (Supporting Information Appendix D). Pregnancy outcomes captured via ICD‐10 codes included operative delivery, cesarean delivery, preterm birth, preterm premature rupture of membranes with preterm delivery, gestational hypertension, preeclampsia, hemolysis elevated liver enzymes low platelets syndrome, eclampsia, and GDM (Supporting Information Appendix E).

Categorical variables were compared using chi‐square tests and continuous variables with two‐sample *t*‐tests. Statistical significance was set at *p* < 0.05. Logistic regression was used to assess risk factors associated with early diabetes screening, with results reported as odds ratios with 95% confidence intervals (CIs) and adjusted for diabetes risk factors to minimize confounding.

Because predictors for early diabetes screening fell into two conceptually distinct groups, we constructed two separate multivariable models for clarity and interpretability. The first model evaluated demographic characteristics associated with receipt of early screening and adjusted for other demographic covariates. The second model evaluated clinical risk factors for diabetes and included adjustment for maternal age and a composite SDOH variable. Race was excluded from the diabetes risk factor model due to a substantial proportion of unknown race in the dataset. Parity was not included because number of prior births cannot be reliably identified in claims data.

Because the predictors evaluated in the multivariable regression models represent parameters within a prespecified analytic model rather than independent hypothesis tests, formal adjustment for multiple comparisons was not applied. Pregnancy outcomes were prespecified and clinically related; therefore statistical significance was evaluated using a two‐sided *α* of 0.05.

The statistical analysis software used was SAS 9.4 (2024).

## 3 RESULTS

**FIGURE 1:**
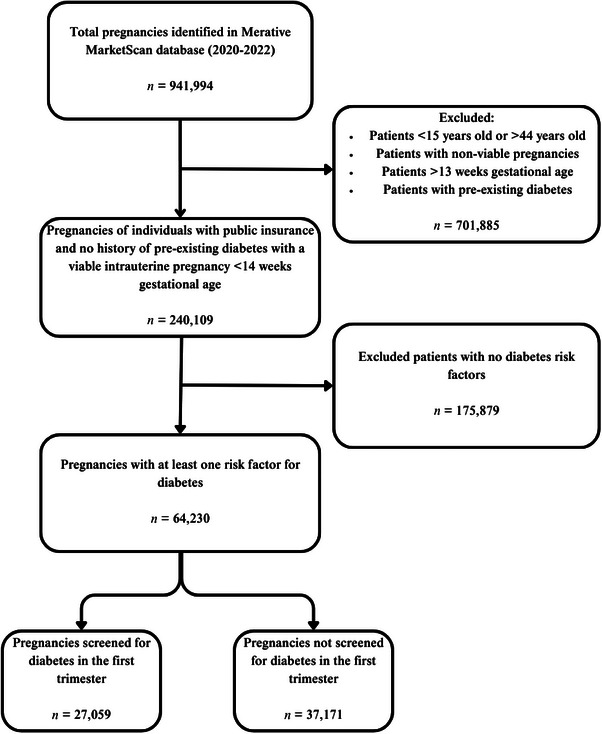
Flow diagram of cohort selection.

A total of 240,109 publicly insured individuals with no history of preexisting diabetes with a viable intrauterine pregnancy <14 weeks gestational age were identified with 64,230 (26.7%) classified as high‐risk for DM. Of these, 27,059 (42.1%) underwent early screening and 37,171 (57.7%) did not (Figure 1). Differences in demographics, social determinants, and risk factors are shown in Table 1. Age and race distributions were similar in both groups. One third of the patients in each cohort identified as Black and 6%–10% identified as Hispanic. Only a small fraction of patients had any documented SDOH ICD‐10 codes, with only 3% in each group having at least one.

**TABLE 1:** Demographic and clinical characteristics of patients in Merative MarketScan with public insurance from January 2020 to December 2022, with a live intrauterine pregnancy and no history of pregestational diabetes, who met criteria for first‐trimester diabetes screening.

Maternal demographic characteristics	Received early diabetes screening (*N* = 27,059)	Did not receive early diabetes screening (*N* = 37,171)	*p* value
Age	28.1 (5.6)	27.8 (5.5)	<0.001
Race			
White	12,083 (44.7)	17,882 (48.1)	<0.001
Black/African American	9539 (35.3)	13,573 (36.5)	
Hispanic	2724 (10.1)	2225 (6.0)	
Other	1207 (4.5)	1415 (3.8)	
Unknown	1506 (5.6)	2076 (5.6)	
Social Determinants of Health (composite)	980 (3.6)	1212 (3.3)	0.0128
**Risk factors for developing diabetes**			
Prepregnancy BMI (≥30)	17,584 (65.0)	16,252 (43.7)	<0.001
Prior history of gestational diabetes	2148 (7.9)	826 (2.2)	<0.001
Hypertension	4357 (16.1)	6083 (16.4)	0.3723
Family history of diabetes	949 (3.5)	693 (1.9)	<0.001
Polycystic ovary syndrome	812 (3.0)	875 (2.4)	<0.001
Hyperlipidemia	655 (2.4)	645 (1.7)	<0.001
Prior pregnancy with macrosomia or large for gestational age infant	8712 (32.2)	17,284 (46.5)	<0.001
Lack of physical exercise/physical inactivity	3 (0.0)	0 (0.0)	–
Insulin resistance	2552 (9.4)	542 (1.5)	<0.001
Cardiovascular disease	626 (2.3)	1444 (3.9)	<0.001
HIV	128 (0.5)	287 (0.8)	0.002

Abbreviations: BMI, body mass index; HIV, human immunodeficiency virus.

Individuals that received early DM screening had higher rates of prepregnancy obesity (65.0% vs. 43.7%, *p* < 0.001), history of GDM (7.9% vs. 2.2%, *p* < 0.001), and lower rates of history of macrosomia or LGA infant (32.2% vs. 46.5%, *p* < 0.001). There were, however, similar rates of hypertension (16.1% vs. 16.4%, *p* = 0.34) in both the screened and unscreened groups (Table 1).

Compared to patients identified as White, Hispanic patients were more likely to be screened early for DM (adjusted odds ratio [aOR] 1.81 95% CI 1.71–1.93) (Table 2). Patients with adverse SDOH factors moderately increased the odds of early DM screening (aOR 1.11 95% CI 1.02–1.21) (Table 2).

**TABLE 2:** Logistic regression for early diabetes screening based on demographic characteristics.

Variable	Unadjusted OR (95% CI)	Adjusted OR^a^ (95% CI)
Age	1.01 (1.01, 1.01)	1.01 (1.01, 1.01)
Race		
White	Ref.	Ref.
Black/African American	1.04 (1.00, 1.07)	1.03 (0.99, 1.06)
Hispanic	1.81 (1.71, 1.93)	1.81 (1.71, 1.93)
Other	1.26 (1.17, 1.37)	1.25 (1.15, 1.35)
Unknown	1.07 (1.00, 1.15)	1.06 (0.99, 1.14)
Social determinants of health (composite)	1.16 (1.02, 1.22)	1.11 (1.02, 1.21)

Abbreviations: CI, confidence interval; OR, odds ratio.

^a^ Adjusted for age, Black/African American, Hispanic, other, unknown, and social determinants of health (composite).

Risk factors most associated with early screening included insulin resistance (aOR 9.47, 95% CI 8.58–10.47), prior GDM (aOR 5.89, 95% CI 5.4–6.43), family history of DM (aOR 3.34, 95% CI 3–3.71), obesity (aOR 3.17, 95% CI 3.04–3.31), hyperlipidemia (aOR 1.59, 95% CI 1.41–1.8), and PCOS (aOR 1.59, 95% CI 1.43–1.77) (Table 3). Known risk factors for diabetes, including history of macrosomia or LGA infant, CVD, lack of physical activity, and HIV were not associated with likelihood of early screening (Table 3).

**TABLE 3:** Logistic regression for early diabetes screening based on risk factors for diabetes.

Variable	Unadjusted OR (95% CI)	Adjusted OR^a^ (95% CI)
Age	1.01 (1.01, 1.01)	1.01 (1.01, 1.01)
Prepregnancy BMI (≥30)	2.39 (2.31, 2.47)	3.17 (3.04, 3.31)
Prior history of gestational diabetes	3.79 (3.50, 4.12)	5.89 (5.4, 6.43)
Hypertension	0.98 (0.94, 1.02)	1.25 (1.19, 1.31)
Family history of diabetes	1.91 (1.73, 2.11)	3.34 (3, 3.71)
Polycystic ovary syndrome	1.28 (1.17, 1.41)	1.59 (1.43, 1.77)
Hyperlipidemia	1.41 (1.26, 1.57)	1.59 (1.41, 1.8)
Prior pregnancy with macrosomia or large for gestational age infant	0.55 (0.53, 0.57)	1.02 (0.98, 1.06)
Lack of physical exercise/physical inactivity	—	—
Insulin resistance	7.04 (6.41, 7.73)	9.47 (8.58, 10.47)
Cardiovascular disease	0.59 (0.53, 0.65)	0.92 (0.83, 1.02)
HIV	0.61 (0.50, 0.75)	0.97 (0.78, 1.21)
Social determinants of health (composite)	1.16 (1.02, 1.22)	1.11 (1.01, 1.21)

Abbreviations: BMI, body mass index; CI, confidence interval; HIV, human immunodeficiency virus; OR, odds ratio.

^a^ Adjusted for the different diabetes risk factors listed in the table: age, prepregnancy BMI ≥30, prior history of gestational diabetes, hypertension, family history of diabetes, polycystic ovary syndrome, hyperlipidemia, prior pregnancy with macrosomia or a large‐for‐gestational‐age infant, lack of physical exercise or physical inactivity, insulin resistance, cardiovascular disease, HIV, and social determinants of health (composite).

GDM was diagnosed in 22.8% of patients who underwent early screening and in 12.9% of patients who did not (*p* < 0.001) (Table 4). Patients who received early screening had higher rates of gestational hypertension (21.2% vs. 19.7%, *p* < 0.001) and other hypertensive disorders of pregnancy (17.4% vs. 15.6%, *p* < 0.001). In addition, they had higher rates of cesarean delivery (16.8% vs. 16.1%, *p* = 0.02), and decreased rates of preterm delivery (6.0% vs. 6.7%, *p* = 0.002) (Table 4).

**TABLE 4:** Comparison of pregnancy outcomes among all patients who met criteria for early diabetes screening.

	Received early diabetes screening (*N* = 27,059)	Did not receive early diabetes screening (*N* = 37,171)	*p* value
Operative delivery	345 (1.3)	541 (1.5)	0.0529
Cesarean delivery	4539 (16.8)	5984 (16.1)	0.0223
Preterm delivery	1637 (6)	2478 (6.7)	0.0016
Gestational hypertension	5739 (21.2)	7339 (19.7)	<0.001
Preeclampsia/HELLP/eclampsia	4703 (17.4)	5817 (15.6)	<0.001
Gestational diabetes mellitus	6180 (22.8)	4777 (12.9)	<0.001

Abbreviation: HELLP, hemolysis elevated liver enzymes low platelets.

A total of 34,740 early screening tests were performed with the majority using HgbA1c (50.0%), 1‐h GTT (29.4%), and fasting glucose (11.5%) (Table 5).

**TABLE 5:** Frequency of each early diabetes screening strategy in this patient population.

Screening modality	Frequency of use (*n*, % of total tests)
Hemoglobin A1c	17,369 (50.0)
Oral glucose tolerance test (50 g)	10,215 (29.4)
Oral glucose tolerance test (75 g)	207 (0.6)
Oral glucose tolerance test (100 g)	32 (0.1)
Fasting plasma glucose	3980 (11.5)
Fingerstick glucose	862 (2.5)
Other	2075 (6.0)

## 4 DISCUSSION

In our study, fewer than half of publicly insured patients with at least one diabetes risk factor underwent first‐trimester screening for preexisting diabetes. Not all the risk factors prompted screening equally; conditions such as insulin resistance and history of GDM were more likely to prompt early screening, whereas history of macrosomia or LGA infant in previous pregnancy did not. Screening modalities also differed in frequency, with HgbA1c testing being the most common. Finally, patients who underwent early screening had higher rates of adverse pregnancy outcomes.

These findings echo prior work demonstrating that early diabetes screening is inconsistently applied, even among patients with risk factors [20]. That study included patients with and without risk factors, across varied settings and insurance sources. The authors found that public insurance was associated with higher likelihood of early diabetes screening, which contradicts our study findings that may be due to the small sample size and heterogeneous population included in their study [20].

Wilkie et al. conducted a claims‐based study of privately insured patients and investigated early diabetes screening trends in patients with and without risk factors for developing DM [18]. They found that approximately 20% of patients in this cohort underwent early screening and they identified multiple diabetes risk factors that were associated with such screening [18]. Our study found that many of those same risk factors, such as obesity and history of GDM, were also associated with early screening in publicly insured pregnancies. However, in contrast to our study, Wilkie et al. identified a history of GDM as the strongest predictor for early screening among privately insured patients, but our study found that among publicly insured patients, insulin resistance was the strongest predictor for early diabetes screening. Differences in baseline characteristics between their study (privately insured, inclusion of all patients) versus our study (publicly insured, inclusion of only those with at least one risk factor) may explain the variation in findings. Despite these differences, our study also identified that publicly insured patients who received early screening experienced higher rates of adverse pregnancy outcomes.

Our study found that diabetes risk factors did not prompt early screening equally. High risk factors such as obesity, prior history of GDM, family history of preexisting diabetes, hypertension, hyperlipidemia, PCOS, and insulin resistance were linked to increased screening rates. Surprisingly, although strong associations have been well established between history of prior macrosomia/LGA neonate and increased risk of DM [21, 22], these patients had lower than expected rates of early screening. In addition, other risk factors like CVD, physical inactivity, and HIV did not prompt early screening. This may represent a gap in providers correctly identifying risk factors that merit early DM screening.

More adverse pregnancy outcomes, most notably GDM, were noted in the group that received early screening as compared to those not screened. This suggests that patients selected for early screening may be at higher baseline risk for these outcomes; however, we are unable to determine causality.

In our study, HgbA1c was the most commonly used screening tool which reflects similar findings among privately insured patients [18]. This likely reflects its convenience, lack of fasting requirement, and good tolerability during pregnancy compared with the GTT [23]. Although the 1‐h GTT was used in 30% of cases, this period coincided with prior ACOG recommendations supporting early GDM screening [9]. With current guidance discouraging early GDM screening [9], use of this test may continue to decline as HgbA1c use rises.

Substantial underscreening for DM in early pregnancy represents a missed opportunity for intervention, particularly in the first trimester when glucose optimization is most crucial [24]. HgbA1c levels above 6.5% in early pregnancy are associated with increased risk of congenital anomalies, affecting fetal cardiac, renal, and central nervous systems [25]. Congenital anomalies impose significant health‐care costs with one study estimating first‐year surgical costs at $7.7 billion, largely from cardiovascular and gastrointestinal conditions [26], while another estimated that birth‐defect related hospitalization in the United States cost $22.2 billion in 2019 [27]. Timely diagnosis and management of pregestational DM could improve outcomes and reduce costs.

This study highlights a large gap in screening within a population at high risk of undiagnosed preexisting diabetes [4, 28]. This issue is part of a larger pattern of health‐care system shortcomings within the United States, as half of nonpregnant individuals who meet criteria for diabetes screening actually receive such screening according to a recent study by Ali et al [16]. Despite clear evidence that undiagnosed diabetes, whether in a pregnant or nonpregnant individual, carries substantial individual health and economic consequences, screening remains inconsistently implemented, even among patients with recognized risk factors. This may be explained by health‐care system failures to adequately capture at‐risk populations, followed by missed opportunities to identify and screen high risk individuals. Relying on providers alone to identify and screen patients may be unsustainable within an already strained health‐care system [29].

This suggests that universal screening should be considered as it may be a more equitable approach to identifying diabetes in the first trimester of pregnancy across all populations. In early 2025, the American Diabetes Association (ADA) argued for universal early screening for DM in populations with increased prevalence of DM risk factors or those with higher rates of undiagnosed diabetes. The ADA publication also highlighted how significant racial and ethnic disparities are seen among patients with undiagnosed DM [30]. Heyborne and Barbour similarly made the case for universal DM screening, citing the rising prevalence of diabetes risk factors in the contemporary US population [11]. They argued that targeted screening adds unnecessary complexity to a test from which most pregnant individuals in the United States, many of which already have an elevated risk for developing DM, may benefit. Supporting this argument, recent data showed that an estimated 52% of the US pregnant population was either overweight or obese [31]. In addition, pregnancy rates among individuals with advanced maternal age (AMA), a known DM risk factor, are increasing with 19% of pregnancies in 2020 occurring in this group [32]. This age group is twice to three times as likely to have undiagnosed DM compared to younger age groups [4]. Additionally, data from 2021 to 2023 showed that approximately 15% of the US population had DM, increasing the likelihood of a family history of DM among pregnant individuals [33]. The rising prevalence of DM risk factors among the US pregnant population, persistently high rates of undiagnosed DM, and the availability of a practical screening test such as the HgbA1c, collectively support a shift toward universal early pregnancy screening for DM.

Although there is debate around optimal early pregnancy diabetes screening strategies, it is important to note that current US guidance recommends risk‐based rather than universal early screening for diabetes. Our findings should be interpreted as descriptive of real‐world screening patterns rather than as a direct measure of adherence to a universally endorsed standard of care.

Future research should investigate the obstacles that prevent publicly insured patients from receiving early screening, including system‐level barriers, inconsistent provider recognition of risk factors, limited resources in practices, and policy or coverage changes within public insurance. Geographic variations should be investigated to identify region‐specific barriers or opportunities for intervention. Finally, cost‐effectiveness analyses are needed to determine whether universal early screening confers meaningful clinical and economic value.

The strengths of this study include the large sample size from a nationwide database, and data originating from different practice settings, which make our findings more generalizable. To our knowledge, this is the largest study evaluating the rates of early diabetes screening in a contemporary cohort of publicly insured pregnant patients in the United States and addresses an important gap in the literature.

Limitations include possible missed diagnoses or erroneous inclusion or exclusion of patients due to the utilization of ICD‐10 and CPT codes. It is also impossible to ascertain how different institutions or providers defined diagnoses like “preeclampsia” or “macrosomia,” as more granular data of this nature are unavailable when utilizing a claims‐based database. This is particularly important for “insulin resistance,” which emerged as one of the strongest predictors of early screening. However, it remains unclear whether this diagnostic code was consistently applied to reflect true clinical insulin resistance, as the underlying signs or symptoms prompting its use were not captured in the Merative Marketscan database. However, prior studies comparing the gold standard of medical record documentation and diagnostic codes for common obstetric diagnoses, including those in this study, show high sensitivity, specificity, and predictive value [34].

As this analysis relied on administrative claims data, identification of screening was dependent on accurate CPT coding, which is another limitation of this study. Point‐of‐care testing, bundled laboratory panels, or services not submitted for reimbursement may not have been captured, potentially resulting in underestimation of true screening rates.

In addition, geographic information was unavailable, so we are unable to comment on geographical differences in early screening. Knowledge regarding geographical differences in practice patterns is valuable as it highlights region‐ and community‐specific challenges faced by patients [29] and can then help direct future research efforts and interventions to areas with the greatest need. In addition, due to the study design, we could not establish causality between screening and pregnancy outcomes, and the study was not specifically powered to evaluate race and ethnicity‐based differences in screening.

Finally, the study period overlaps with the COVID‐19 pandemic which may have temporarily influenced clinical practice patterns. Noticeable changes included reduced in‐person visits [35], increased reliance on telehealth, and potential laboratory access limitations [36]. However, studies centered on that time period have shown that screening strategies were minimally affected during the COVID‐19 pandemic [37, 38, 39]. With regard to diabetes in pregnancy, one Canadian study found that more than 95% of pregnancies from January 2020 to December 2021 were screened for GDM [38]. Another Canadian study found a modest increase in GDM screening among pregnant populations during the COVID‐19 pandemic [37]. In the United States, a study focused on nonpregnant individuals found only a 4% significant decrease in diabetes screening from 2019 to 2021 [39]. The marginal changes in diabetes screening practices across these different patient populations support that the significant findings of our study cannot be solely explained by the pandemic era changes in clinical care. Although prior studies suggest that diabetes screening practices were only modestly affected during this period, pandemic‐related disruptions may have contributed to under‐ or overestimation of early screening rates and may limit generalizability to post‐pandemic care environments.

## 5 CONCLUSIONS

In conclusion, almost 60% of high‐risk individuals with public insurance in the United States did not receive first‐trimester diabetes screening. Given the rising prevalence of obesity, chronic medical conditions, and AMA, known factors that increase the likelihood of undiagnosed diabetes, further research is needed to address disparities in screening, improve early detection among high‐risk populations, and evaluate whether universal screening may be warranted. Improving early pregnancy diabetes screening rates may require actionable changes on multiple fronts including provider education reminders on risk‐factor identification, integration of screening reminders in electronic health record systems for eligible patients, standardized screening protocols, and continued examination of universal screening strategies to promote equitable screening and disease detection across all populations.

## AUTHOR CONTRIBUTIONS

**Benjamin Koethe**: Data curation; formal analysis; writing—review and editing. **Sebastian Z. Ramos**: Conceptualization; writing—review and editing; data curation; funding acquisition. **Zeinab Kassem**: Conceptualization; methodology; data curation; writing—original draft. **Mohak Mhatre**: Conceptualization; writing—review and editing; data curation.

## CONFLICT OF INTEREST STATEMENT

The authors declare no conflicts of interest.

## Supporting information



Supporting Information

## Data Availability

The data that support the findings of this study are available from Merative MarketScan. Restrictions apply to the availability of these data, which were used under license for this study. Access to the data may be obtained directly from Merative with the permission of the data provider.
