# Supplementary material for: First-trimester preexisting diabetes screening in Medicaid beneficiaries
Source: Pregnancy (Hoboken). Author manuscript; Available in PMC 2026 May 30. (PMC13220985; doi:10.1002/pmf2.70290)
Supplement: Appendix [file NIHMS2175759-supplement-Appendix.docx]

**Appendix:**

Appendix A: ICD codes used to identify patients who meet the inclusion criteria for the study.

| **ICD code** | **Diagnosis** |
| --- | --- |
| Z3A.0 | Weeks of gestation of pregnancy, unspecified or less than 10 weeks |
| Z3A.01 | Less than 8 weeks gestation of pregnancy |
| Z3A.08 | 8 weeks gestation of pregnancy |
| Z3A.09 | 9 weeks gestation of pregnancy |
| Z3A.10 | 10 weeks gestation of pregnancy |
| Z3A.11 | 11 weeks gestation of pregnancy |
| Z3A.12 | 12 weeks gestation of pregnancy |
| Z3A.13 | 13 weeks gestation of pregnancy |
| Z34.01 | Encounter for supervision of normal first pregnancy, first trimester |
| Z34.81 | Encounter for supervision of other normal pregnancy, first trimester |
| Z34.91 | Encounter for supervision of normal pregnancy, unspecified, first trimester |
| O09.91 | Supervision of high risk pregnancy, unspecified, first trimester |
| O09.11 | Supervision of pregnancy with history of ectopic pregnancy, first trimester |
| O09.A1 | Supervision of pregnancy with history of molar pregnancy, first trimester |
| O09.211 | Supervision of pregnancy with history of pre-term labor, first trimester |
| O09.291 | Supervision of pregnancy with other poor reproductive or obstetric history, first trimester |
| O09.31 | Supervision of pregnancy with insufficient antenatal care, first trimester |
| O09.41 | Supervision of pregnancy with grand multiparity, first trimester |
| O09.511 | Supervision of elderly primigravida, first trimester |
| O09.521 | Supervision of elderly multigravida, first trimester |
| O09.611 | Supervision of young primigravida, first trimester |
| O09.621 | Supervision of young multigravida, first trimester |
| O09.71 | Supervision of high risk pregnancy due to social problems, first trimester |
| O09.811 | Supervision of pregnancy resulting from assisted reproductive technology, first trimester |
| O09.821 | Supervision of pregnancy with history of in utero procedure during previous pregnancy, first trimester |
| O09.891 | Supervision of other high risk pregnancies, first trimester |
| O09.91 | Supervision of high risk pregnancy, unspecified, first trimester |
| O09.A1 | Supervision of pregnancy with history of molar pregnancy, first trimester |

Appendix B: ICD codes used to identify diabetes risk factors in this cohort.

| **ICD code** | **Diagnosis** |
| --- | --- |
| Z86.32 | History of Gestational Diabetes |
| O10 | Pre-existing hypertension complicating pregnancy, childbirth and the puerperium |
| O10.0 | Pre-existing essential hypertension complicating pregnancy, childbirth and the puerperium |
| O10.01 | Pre-existing essential hypertension complicating pregnancy |
| O10.011 | Pre-existing essential hypertension complicating pregnancy, first trimester |
| O10.019 | Pre-existing essential hypertension complicating pregnancy, unspecified trimester |
| O10.1 | Pre-existing hypertensive heart disease complicating pregnancy, childbirth and the puerperium |
| O10.11 | Pre-existing hypertensive heart disease complicating pregnancy |
| O10.111 | Pre-existing hypertensive heart disease complicating pregnancy, first trimester |
| O10.119 | Pre-existing hypertensive heart disease complicating pregnancy, unspecified trimester |
| O10.2 | Pre-existing hypertensive chronic kidney disease complicating pregnancy, childbirth and the puerperium |
| O10.21 | Pre-existing hypertensive chronic kidney disease complicating pregnancy |
| O10.211 | Pre-existing hypertensive chronic kidney disease complicating pregnancy, first trimester |
| O10.219 | Pre-existing hypertensive chronic kidney disease complicating pregnancy, unspecified trimester |
| O10.3 | Pre-existing hypertensive heart and chronic kidney disease complicating pregnancy, childbirth and the puerperium |
| O10.31 | Pre-existing hypertensive heart and chronic kidney disease complicating pregnancy |
| O10.311 | Pre-existing hypertensive heart and chronic kidney disease complicating pregnancy, first trimester |
| O10.319 | Pre-existing hypertensive heart and chronic kidney disease complicating pregnancy, unspecified trimester |
| O10.4 | Pre-existing secondary hypertension complicating pregnancy, childbirth and the puerperium |
| O10.41 | Pre-existing secondary hypertension complicating pregnancy, childbirth and the puerperium |
| O10.411 | Pre-existing secondary hypertension complicating pregnancy, first trimester |
| O10.419 | Pre-existing secondary hypertension complicating pregnancy, unspecified trimester |
| O10.9 | Unspecified pre-existing hypertension complicating pregnancy, childbirth and the puerperium |
| O10.91 | Unspecified pre-existing hypertension complicating pregnancy, childbirth and the puerperium |
| O10.911 | Unspecified pre-existing hypertension complicating pregnancy, first trimester |
| O10.919 | Unspecified pre-existing hypertension complicating pregnancy, unspecified trimester |
| I10 | Essential (primary) hypertension |
| I11 | Hypertensive heart disease |
| I11.0 | Hypertensive heart disease with heart failure |
| I11.9 | Hypertensive heart disease without heart failure |
| I12 | Hypertensive chronic kidney disease |
| I12.0 | Hypertensive chronic kidney disease with stage 5 chronic kidney disease or end stage renal disease |
| I12.9 | Hypertensive chronic kidney disease with stage 1 through stage 4 chronic kidney disease, or unspecified chronic kidney disease |
| I13 | Hypertensive heart and chronic kidney disease |
| I13.0 | Hypertensive heart and chronic kidney disease with heart failure and stage 1 through stage 4 chronic kidney disease, or unspecified chronic kidney disease |
| I13.1 | Hypertensive heart and chronic kidney disease without heart failure |
| I13.10 | Hypertensive heart and chronic kidney disease without heart failure, with stage 1 through stage 4 chronic kidney disease, or unspecified chronic kidney disease |
| I13.11 | Hypertensive heart and chronic kidney disease without heart failure, with stage 5 chronic kidney disease, or end stage renal disease |
| I13.2 | Hypertensive heart and chronic kidney disease with heart failure and with stage 5 chronic kidney disease, or end stage renal disease |
| I15 | Secondary hypertension |
| I15.0 | Renovascular hypertension |
| I15.1 | Hypertension secondary to other renal disorders |
| I15.2 | Hypertension secondary to endocrine disorders |
| I15.8 | Other secondary hypertension |
| I15.9 | Secondary hypertension, unspecified |
| Z83.3 | Family History of Diabetes |
| E28.2 | Polycystic Ovarian Syndrome (PCOS) |
| O99.21 | Obesity |
| O99.210 | Obesity complicating pregnancy, unspecified trimester |
| O99.211 | Obesity complicating pregnancy, first trimester |
| E66 | Overweight and obesity |
| E66.0 | Obesity due to excess calories |
| E66.01 | Morbid (severe) obesity due to excess calories |
| E66.09 | Other obesity due to excess calories |
| E66.1 | Drug-induced obesity |
| E66.2 | Morbid (severe) obesity with alveolar hypoventilation |
| E66.3 | Overweight |
| E66.8 | Other obesity |
| E66.9 | Obesity, unspecified |
| E78 | Disorders of lipoprotein metabolism and other lipidemias |
| E78.0 | Pure hypercholesterolemia |
| E78.00 | Pure hypercholesterolemia, unspecified |
| E78.01 | Familial hypercholesterolemia |
| E78.1 | Pure hyperglyceridemia |
| E78.2 | Mixed hyperlipidemia |
| E78.3 | Hyperchylomicronemia |
| E78.4 | Other hyperlipidemia |
| E78.41 | Elevated Lipoprotein(a) |
| E78.49 | Other hyperlipidemia |
| E78.5 | Hyperlipidemia, unspecified |
| E78.6 | Lipoprotein deficiency |
| E78.7 | Disorders of bile acid and cholesterol metabolism |
| E78.70 | Disorder of bile acid and cholesterol metabolism, unspecified |
| E78.71 | Barth syndrome |
| E78.72 | Smith-Lemli-Opitz syndrome |
| E78.79 | Other disorders of bile acid and cholesterol metabolism |
| E78.81 | Lipoid dermatoarthritis |
| E78.8 | Other disorders of lipoprotein metabolism |
| E78.89 | Other lipoprotein metabolism disorders |
| E78.9 | Disorder of lipoprotein metabolism, unspecified |
| P08 | Disorders of newborn related to long gestation and high birth weight |
| P08.0 | Exceptionally large newborn baby |
| P08.1 | Other heavy for gestational age newborn |
| P08.2 | Late newborn, not heavy for gestational age |
| P08.21 | Post-term newborn |
| P08.22 | Prolonged gestation of newborn |
| O09.29 | Supervision of pregnancy with other poor reproductive or obstetric history |
| O09.291 | Supervision of pregnancy with other poor reproductive or obstetric history, first trimester |
| O09.292 | Supervision of pregnancy with other poor reproductive or obstetric history, second trimester |
| O09.293 | Supervision of pregnancy with other poor reproductive or obstetric history, third trimester |
| O09.299 | Supervision of pregnancy with other poor reproductive or obstetric history, unspecified trimester |
| Z87.59 | Personal history of other complications of pregnancy, childbirth and the puerperium |
| O36.61 | Maternal care for excessive fetal growth, first trimester |
| O36.62 | Maternal care for excessive fetal growth, second trimester |
| O36.63 | Maternal care for excessive fetal growth, third trimester |
| Z72. 3 | Lack of physical exercise / Physical inactivity |
| E88.81 | Insulin resistance |
| R73 | Elevated blood glucose level |
| R73.0 | Abnormal glucose |
| R73.01 | Impaired fasting glucose |
| R73.02 | Impaired glucose tolerance (oral) |
| R73.03 | Prediabetes |
| R73.09 | Other abnormal glucose |
| R73.9 | Hyperglycemia, unspecified |
| E88.8 | Other specified metabolic disorders |
| E88.81 | Metabolic syndrome |
| E88.89 | Other specified metabolic disorders |
| E88.9 | Metabolic disorder, unspecified |
| O99.4 | Diseases of the circulatory system complicating pregnancy, childbirth and the puerperium |
| O99.41 | Diseases of the circulatory system complicating pregnancy |
| O99.419 | Diseases of the circulatory system complicating pregnancy, unspecified trimester |
| O98.7 | Human immunodeficiency virus [HIV] disease complicating pregnancy, childbirth, and the puerperium: This is the main code for indicating HIV infection during pregnancy. |
| O98.71 | Human immunodeficiency virus [HIV] disease complicating pregnancy |
| O98.711 | Human immunodeficiency virus [HIV] disease complicating pregnancy, first trimester |
| O98.719 | Human immunodeficiency virus [HIV] disease complicating pregnancy, unspecified trimester |
| B20 | Human immunodeficiency virus [HIV] disease: This is the code for HIV infection in general. |
| Z21 | Asymptomatic human immunodeficiency virus [HIV] infection status: This code is used when the patient is known to be HIV positive but does not have any symptoms of HIV-related illness. |
| O98.714 | Human immunodeficiency virus [HIV] disease complicating pregnancy, childbirth, and the puerperium, antepartum condition or complication: This is a more specific code for indicating HIV infection during pregnancy in the antepartum period. |
| O98.714 | Human immunodeficiency virus [HIV] disease complicating pregnancy, childbirth, and the puerperium, antepartum condition or complication: This is a more specific code for indicating HIV infection during pregnancy in the antepartum period. |

Appendix C: ICD codes used to identify social determinants of health.

| **ICD code** | **Diagnosis** |
| --- | --- |
| Z59 | Problems related to housing and economic circumstances |
| Z59.0 | Homelessness |
| Z59.00 | Homelessness unspecified |
| Z59.01 | Sheltered homelessness |
| Z59.02 | Unsheltered homelessness |
| Z59.1 | Inadequate housing |
| Z59.10 | Inadequate housing, unspecified |
| Z59.11 | Inadequate housing environmental temperature |
| Z59.12 | Inadequate housing utilities |
| Z59.19 | Other inadequate housing |
|  |  |
| Z59.4 | Lack of adequate food |
| Z59.41 | Food insecurity |
| Z59.48 | Other specified lack of adequate food |
| Z59.5 | Extreme poverty |
| Z59.6 | Low income |
|  |  |
| Z59.8 | Other problems related to housing and economic circumstances |
| Z59.81 | Housing instability, housed |
| Z59.811 | Housing instability, housed, with risk of homelessness |
| Z59.812 | Housing instability, housed, homelessness in past 12 months |
| Z59.819 | Housing instability, housed unspecified |
| Z59.82 | Transportation insecurity |
| Z59.86 | Financial insecurity |
| Z59.87 | Material hardship due to limited financial resources, not elsewhere classified |
| Z59.89 | Other problems related to housing and economic circumstances |
| Z59.9 | Problem related to housing and economic circumstances, unspecified |
|  |  |
| Z55 | Problems related to education and literacy |
| Z55.0 | Illiteracy and low-level literacy |
| Z55.1 | Schooling unavailable and unattainable |
| Z55.2 | Failed school examinations |
| Z55.3 | Underachievement in school |
| Z55.4 | Educational maladjustment and discord with teachers and classmates |
| Z55.5 | Less than a high school diploma |
| Z55.6 | Problems related to health literacy |
| Z55.8 | Other problems related to education and literacy |
| Z55.9 | Problems related to education and literacy, unspecified |
|  |  |
| Z56 | Problems related to employment and unemployment |
| Z56.0 | Unemployment, unspecified |
| Z56.1 | Change of job |
| Z56.2 | Threat of job loss |
| Z56.3 | Stressful work schedule |
| Z56.4 | Discord with boss and workmates |
| Z56.5 | Uncongenial work environment |
| Z56.6 | Other physical and mental strain related to work |
| Z56.8 | Other problems related to employment |
| Z56.81 | Sexual harassment on the job |
| Z56.82 | Military deployment status |
| Z56.89 | Other problems related to employment |
| Z56.9 | Unspecified problems related to employment |
|  |  |
| Z57 | Occupational exposure to risk factors |
| Z57.0 | Occupational exposure to noise |
| Z57.1 | Occupational exposure to radiation |
| Z57.2 | Occupational exposure to dust |
| Z57.3 | Occupational exposure to other air contaminants |
| Z57.31 | Occupational exposure to environmental tobacco smoke |
| Z57.39 | Occupational exposure to other air contaminants |
| Z57.4 | Occupational exposure to toxic agents in agriculture |
| Z57.5 | Occupational exposure to toxic agents in other industries |
| Z57.6 | Occupational exposure to extreme temperature |
| Z57.7 | Occupational exposure to vibration |
| Z57.8 | Occupational exposure to other risk factors |
| Z57.9 | Occupational exposure to unspecified risk factor |
|  |  |
| Z58 | Problems related to physical environment |
| Z58.6 | Inadequate drinking-water supply |
| Z58.8 | Other problems related to physical environment |
| Z58.81 | Basic services unavailable in physical environment |
| Z58.89 | Other problems related to physical environment |
|  |  |
| Z59 | Problems related to housing and economic circumstances |
| Z59.0 | Homelessness |
| Z59.00 | Homelessness unspecified |
| Z59.01 | Sheltered homelessness |
|  |  |
| Z59.02 | Unsheltered homelessness |
| Z59.1 | Inadequate housing |
| Z59.10 | Inadequate housing, unspecified |
| Z59.11 | Inadequate housing environmental temperature |
| Z59.12 | Inadequate housing utilities |
| Z59.19 | Other inadequate housing |
| Z59.2 | Discord with neighbors, lodgers and landlord |
| Z59.3 | Problems related to living in residential institution |
| Z59.4 | Lack of adequate food |
| Z59.41 | Food insecurity |
| Z59.48 | Other specified lack of adequate food |
| Z59.5 | Extreme poverty |
| Z59.6 | Low income |
| Z59.7 | Insufficient social insurance and welfare support |
| Z59.8 | Other problems related to housing and economic circumstances |
| Z59.81 | Housing instability, housed |
| Z59.811 | Housing instability, housed, with risk of homelessness |
| Z59.812 | Housing instability, housed, homelessness in past 12 months |
| Z59.819 | Housing instability, housed unspecified |
| Z59.82 | Transportation insecurity |
| Z59.86 | Financial insecurity |
| Z59.87 | Material hardship due to limited financial resources, not elsewhere classified |
| Z59.89 | Other problems related to housing and economic circumstances |
| Z59.9 | Problem related to housing and economic circumstances, unspecified |
|  |  |
| Z60 | Problems related to social environment |
| Z60.0 | Problems of adjustment to life-cycle transitions |
| Z60.2 | Problems related to living alone |
| Z60.3 | Acculturation difficulty |
| Z60.4 | Social exclusion and rejection |
| Z60.5 | Target of (perceived) adverse discrimination and persecution |
| Z60.8 | Other problems related to social environment |
| Z60.9 | Problem related to social environment, unspecified |
|  |  |
| Z62 | Problems related to upbringing |
| Z62.0 | Inadequate parental supervision and control |
| Z62.1 | Parental overprotection |
| Z62.2 | Upbringing away from parents |
| Z62.21 | Child in welfare custody |
| Z62.22 | Institutional upbringing |
| Z62.23 | Child in custody of non-parental relative |
| Z62.24 | Child in custody of non-relative guardian |
| Z62.29 | Other upbringing away from parents |
| Z62.3 | Hostility towards and scapegoating of child |
| Z62.6 | Inappropriate (excessive) parental pressure |
| Z62.8 | Other specified problems related to upbringing |
| Z62.81 | Personal history of abuse in childhood |
| Z62.810 | Personal history of physical and sexual abuse in childhood |
| Z62.811 | Personal history of psychological abuse in childhood |
| Z62.812 | Personal history of neglect in childhood |
| Z62.813 | Personal history of forced labor or sexual exploitation in childhood |
| Z62.814 | Personal history of child financial abuse |
| Z62.815 | Personal history of intimate partner abuse in childhood |
| Z62.819 | Personal history of unspecified abuse in childhood |
| Z62.82 | Parent-child conflict |
| Z62.820 | Parent-biological child conflict |
| Z62.821 | Parent-adopted child conflict |
| Z62.822 | Parent-foster child conflict |
| Z62.823 | Parent-step child conflict |
| Z62.83 | Non-parental relative or guardian-child conflict |
| Z62.831 | Non-parental relative-child conflict |
| Z62.832 | Non-relative guardian-child conflict |
| Z62.833 | Group home staff-child conflict |
| Z62.89 | Other specified problems related to upbringing |
| Z62.890 | Parent-child estrangement NEC |
| Z62.891 | Sibling rivalry |
| Z62.892 | Runaway [from current living environment] |
| Z62.898 | Other specified problems related to upbringing |
| Z62.9 | Problem related to upbringing, unspecified |
|  |  |
| Z63 | Other problems related to primary support group, including family circumstances |
| Z63.0 | Problems in relationship with spouse or partner |
| Z63.1 | Problems in relationship with in-laws |
| Z63.3 | Absence of family member |
| Z63.31 | Absence of family member due to military deployment |
| Z63.32 | Other absence of family member |
| Z63.4 | Disappearance and death of family member |
| Z63.5 | Disruption of family by separation and divorce |
| Z63.6 | Dependent relative needing care at home |
| Z63.7 | Other stressful life events affecting family and household |
| Z63.71 | Stress on family due to return of family member from |
| Z63.72 | Alcoholism and drug addiction in family |
| Z63.79 | Other stressful life events affecting family and household |
| Z63.8 | Other specified problems related to primary support group |
| Z63.9 | Problem related to primary support group, unspecified |
|  |  |
| Z64 | Problems related to certain psychosocial circumstances |
| Z64.0 | Problems related to unwanted pregnancy |
| Z64.1 | Problems related to multiparity |
| Z64.4 | Discord with counselors |
|  |  |
| Z65 | Problems related to other psychosocial circumstances |
| Z65.0 | Conviction in civil and criminal proceedings without imprisonment |
| Z65.1 | Imprisonment and other incarceration |
| Z65.2 | Problems related to release from prison |
| Z65.3 | Problems related to other legal circumstances |
| Z65.4 | Victim of crime and terrorism |
| Z65.5 | Exposure to disaster, war and other hostilities |
| Z65.8 | Other specified problems related to psychosocial circumstances |
| Z65.9 | Problem related to unspecified psychosocial circumstances |
|  |  |
| O09.7 | Supervision of high risk pregnancy due to social problems |
| O09.70 | Supervision of high risk pregnancy due to social problems, unspecified trimester |
| O09.71 | Supervision of high risk pregnancy due to social problems, first trimester |
| O09.72 | Supervision of high risk pregnancy due to social problems, second trimester |
| O09.73 | Supervision of high risk pregnancy due to social problems, third trimester |
| O09.3 | Supervision of pregnancy with insufficient antenatal care |
| O09.30 | Supervision of pregnancy with insufficient antenatal care, unspecified trimester |
| O09.31 | Supervision of pregnancy with insufficient antenatal care, first trimester |
| O09.32 | Supervision of pregnancy with insufficient antenatal care, second trimester |
| O09.33 | Supervision of pregnancy with insufficient antenatal care, third trimester |

Appendix D: CPT codes used to identify diabetes screening test in this cohort.

| **Screening modality** | **CPT code** |
| --- | --- |
| Hemoglobin A1c | 83036 OR 83037 |
| Oral glucose tolerance test (50g) | 82950 |
| Oral glucose tolerance test (75g) | 82947 + 82950 |
| Oral glucose tolerance test (100g) | 82947 + 82951 |
| Fasting plasma glucose | 82947 |
| Fingerstick glucose | 82962 |

Appendix E: ICD codes used to identify pregnancy outcomes in this cohort.

| **ICD code** | **Diagnosis** |
| --- | --- |
| O11.2 | Pre-existing hypertension with pre-eclampsia, second trimester |
| O11.3 | Pre-existing hypertension with pre-eclampsia, third trimester |
| O11.4 | Pre-existing hypertension with pre-eclampsia, complicating childbirth |
| O11.5 | Pre-existing hypertension with pre-eclampsia, complicating the puerperium |
| O11.9 | Pre-existing hypertension with pre-eclampsia, unspecified trimester |
| O14 | Pre-eclampsia |
| O14.0 | Mild to moderate pre-eclampsia |
| O14.00 | Mild to moderate pre-eclampsia, unspecified trimester |
| O14.02 | Mild to moderate pre-eclampsia, second trimester |
| O14.03 | Mild to moderate pre-eclampsia, third trimester |
| O14.04 | Mild to moderate pre-eclampsia, complicating childbirth |
| O14.05 | Mild to moderate pre-eclampsia, complicating the puerperium |
| O14.1 | Severe pre-eclampsia |
| O14.10 | Severe pre-eclampsia, unspecified trimester |
| O14.12 | Severe pre-eclampsia, second trimester |
| O14.13 | Severe pre-eclampsia, third trimester |
| O14.14 | Severe pre-eclampsia complicating childbirth |
| O14.15 | Severe pre-eclampsia, complicating the puerperium |
| O14.2 | HELLP syndrome |
| O14.20 | HELLP syndrome (HELLP), unspecified trimester |
| O14.22 | HELLP syndrome (HELLP), second trimester |
| O14.23 | HELLP syndrome (HELLP), third trimester |
| O14.24 | HELLP syndrome, complicating childbirth |
| O14.25 | HELLP syndrome, complicating the puerperium |
| O14.9 | Unspecified pre-eclampsia |
| O14.90 | Unspecified pre-eclampsia, unspecified trimester |
| O14.92 | Unspecified pre-eclampsia, second trimester |
| O14.93 | Unspecified pre-eclampsia, third trimester |
| O14.94 | Unspecified pre-eclampsia, complicating childbirth |
| O14.95 | Unspecified pre-eclampsia, complicating the puerperium |
| O15 | Eclampsia |
| O15.0 | Eclampsia complicating pregnancy |
| O15.00 | Eclampsia complicating pregnancy, unspecified trimester |
| O15.02 | Eclampsia complicating pregnancy, second trimester |
| O15.03 | Eclampsia complicating pregnancy, third trimester |
| O15.1 | Eclampsia complicating labor |
| O15.2 | Eclampsia complicating the puerperium |
| O15.9 | Eclampsia, unspecified as to time period |
| O60.1 | Preterm delivery |
| O60.10 | Preterm labor with preterm delivery, unspecified trimester |
| O60.12 | Preterm labor second trimester with preterm delivery second trimester |
| O60.13 | Preterm labor second trimester with preterm delivery third trimester |
| O60.14 | Preterm labor third trimester with preterm delivery third trimester |
| O42.01 | Preterm premature rupture of membranes, onset of labor within 24 hours of rupture |
| O42.012 | Preterm premature rupture of membranes, onset of labor within 24 hours of rupture, second trimester |
| O42.013 | Preterm premature rupture of membranes, onset of labor within 24 hours of rupture, third trimester |
| O42.019 | Preterm premature rupture of membranes, onset of labor within 24 hours of rupture, unspecified trimester |
| O42.11 | Preterm premature rupture of membranes, onset of labor more than 24 hours following rupture |
| O42.112 | Preterm premature rupture of membranes, onset of labor more than 24 hours following rupture, second trimester |
| O42.113 | Preterm premature rupture of membranes, onset of labor more than 24 hours following rupture, third trimester |
| O42.119 | Preterm premature rupture of membranes, onset of labor more than 24 hours following rupture, unspecified trimester |
| O42.91 | Preterm premature rupture of membranes, unspecified as to length of time between rupture and onset of labor |
| O42.912 | Preterm premature rupture of membranes, unspecified as to length of time between rupture and onset of labor, second trimester |
| O42.913 | Preterm premature rupture of membranes, unspecified as to length of time between rupture and onset of labor, third trimester |
| O42.919 | Preterm premature rupture of membranes, unspecified as to length of time between rupture and onset of labor, unspecified trimester |
| O66.41 | Failed attempted vaginal birth after previous cesarean delivery |
| O75.82 | Onset (spontaneous) of labor after 37 completed weeks of gestation but before 39 completed weeks gestation, with delivery by (planned) cesarean section |
| O82 | Encounter for cesarean delivery without indication |
| Z38.01 | Single liveborn infant, delivered by cesarean |
| Z38.31 | Twin liveborn infant, delivered by cesarean |
| Z38.62 | Triplet liveborn infant, delivered by cesarean |
| Z38.64 | Quadruplet liveborn infant, delivered by cesarean |
| Z38.66 | Quintuplet liveborn infant, delivered by cesarean |
| Z38.69 | Other multiple liveborn infant, delivered by cesarean |
| O66.4 | Failed trial of labor |
| O66.40 | Failed trial of labor, unspecified |
| O66.41 | Failed attempted vaginal birth after previous cesarean delivery |
| O66.5 | Attempted application of vacuum extractor and forceps with subsequent cesarean delivery |
| O75.82 | Onset (spontaneous) of labor after 37 completed weeks of gestation but before 39 completed weeks gestation, with delivery by (planned) cesarean section |
| O61 | Failed induction of labor |
| O61.0 | Failed medical induction of labor |
| O61.1 | Failed instrumental induction of labor |
| O61.8 | Other failed induction of labor |
| O61.9 | Failed induction of labor, unspecified |
| O99.892 | Other specified diseases and conditions complicating childbirth (emergency cesarean) |
| O75.9 | Delivery complicated by attempted vacuum extraction and forceps |
| O13 | Gestational [pregnancy-induced] hypertension without significant proteinuria |
| O13.1 | Gestational [pregnancy-induced] hypertension without significant proteinuria, first trimester |
| O13.2 | Gestational [pregnancy-induced] hypertension without significant proteinuria, second trimester |
| O13.3 | Gestational [pregnancy-induced] hypertension without significant proteinuria, third trimester |
| O13.4 | Gestational [pregnancy-induced] hypertension without significant proteinuria, complicating childbirth |
| O13.5 | Gestational [pregnancy-induced] hypertension without significant proteinuria, complicating the puerperium |
| O13.9 | Gestational [pregnancy-induced] hypertension without significant proteinuria, unspecified trimester |
| O24.4 | Gestational Diabetes |
| O24.41 | Gestational diabetes mellitus in pregnancy |
| O24.410 | Gestational diabetes mellitus in pregnancy, diet controlled |
| O24.414 | Gestational diabetes mellitus in pregnancy, insulin controlled |
| O24.415 | Gestational diabetes mellitus in pregnancy, controlled by oral hypoglycemic drugs |
| O24.419 | Gestational diabetes mellitus in pregnancy, unspecified control |
| O24.42 | Gestational diabetes mellitus in childbirth |
| O24.420 | Gestational diabetes mellitus in childbirth, diet controlled |
| O24.424 | Gestational diabetes mellitus in childbirth, insulin controlled |
| O24.425 | Gestational diabetes mellitus in childbirth, controlled by oral hypoglycemic drugs |
| O24.429 | Gestational diabetes mellitus in childbirth, unspecified control |
| O24.43 | Gestational diabetes mellitus in the puerperium |
| O24.430 | Gestational diabetes mellitus in the puerperium, diet controlled |
| O24.434 | Gestational diabetes mellitus in the puerperium, insulin controlled |
| O24.435 | Gestational diabetes mellitus in puerperium, controlled by oral hypoglycemic drugs |
| O24.439 | Gestational diabetes mellitus in the puerperium, unspecified control |
